# Clinical validation of a plasma-based antibody-free LC–MS method for identifying CSF amyloid positivity in mild cognitive impairment

**DOI:** 10.3389/fnagi.2025.1681516

**Published:** 2025-10-10

**Authors:** José Antonio Allué, Leticia Sarasa, Noelia Fandos, Ricardo Gonzalo, Rubén Sabido-Vera, Jorge Loscos, Judith Romero, Adrián Sánchez, Jose Terencio, Jordi A. Matias-Guiu, Gerard Piñol-Ripoll, María Pascual-Lucas

**Affiliations:** ^1^Araclon Biotech-Grifols, Zaragoza, Spain; ^2^Scientific Innovation Office, Grifols S.A., Barcelona, Spain; ^3^Hospital Clínico Universitario San Carlos, Madrid, Spain; ^4^Unitat de Trastorns Cognitius, Hospital Universitari Santa Maria, Lleida, Spain; ^5^Alzheimer's Disease and Other Cognitive Disorders Unit, Neurology Service, Hospital Clínic de Barcelona, Fundació de Recerca Clínic—Institut d'Investigacions Biomèdiques August Pi i Sunyer (IDIBAPS), Barcelona, Spain

**Keywords:** ABtest-MS, Alzheimer’s disease, amyloid-β, Aβ42/Aβ40 ratio, blood biomarkers, validation, mass spectrometry, mild cognitive impairment

## Abstract

**Background:**

The recent approval of monoclonal antibodies for the treatment of Alzheimer’s disease (AD) in several countries has accelerated the need for affordable, simple and scalable methods to identify patients who are eligible for treatment with the new disease-modifying therapies (DMT). Blood-based biomarkers offer less invasive alternatives to established gold standards. We have clinically validated a predictive model combining plasma Aβ42/Aβ40, apolipoprotein E (APOE) genotype and age, in two independent real-world cohorts to identify brain amyloid deposition.

**Methods:**

We conducted a clinical validation study involving 450 patients with mild cognitive impairment (MCI) from two real-world cohorts (HCSC, Madrid, Spain and HUSM, Lleida, Spain). Plasma Aβ42/Aβ40 was measured by ABtest-MS, an antibody-free liquid chromatography-mass spectrometry method. CSF Aβ42/Aβ40 and p-tau181/Aβ42 (gold standards) were quantified with the Lumipulse^®^ platform. The model was trained in the HCSC cohort and validated in the HUSM cohort. Finally, an overall analysis in the combined population was performed. A dual cutoff approach was used to classify the patients as positive or negative. Statistical analysis included bootstrap resampling and model calibration.

**Results:**

In the HCSC, HUSM, external validation and combined analysis, AUCs were 0.89 (95% confidence intervals-CI: 0.84–0.93), 0.88 (0.84–0.93), 0.88 (0.83–0.92) and 0.88 (0.84–0.91), with corresponding accuracies of 82.3, 81.6, 82.3, and 81.1%, respectively. After the combined analysis, positive and negative predictive values (PPV and NPV) were established at 87.5%, resulting in cutoff values of 0.30 and 0.67 for the likelihood of amyloid negativity and positivity, respectively, for a prevalence of 62%. Probability values lower than 0.30 indicate low probability of brain amyloid deposition, while values greater than 0.67 indicate high probability. Less than 28% of the participants fell into the intermediate zone. Additional cutoffs were derived for different prevalence values. Predictive model calibration showed excellent agreement with observed data, confirming accurate predictions (slope = 0.98, intercept = −0.01).

**Conclusion:**

This predictive model has demonstrated high accuracy for the identification of brain amyloid deposition in patients with MCI. Derived cutoffs enabled over 70% reduction in invasive testing, supporting efficient and cost-effective identification of candidates for DMTs.

## 1 Introduction

As the leading cause of dementia worldwide, Alzheimer’s disease (AD) continues to rise in incidence (Alzheimer’s Association, 2024), placing an increasing strain on healthcare systems, which struggle to meet the growing demand.

Recently, several countries have granted regulatory approval to the monoclonal antibodies lecanemab and donanemab for the treatment of early symptomatic AD patients with confirmed amyloid-beta (Aβ) pathology (van Dyck et al., 2023; Sims et al., 2023). These disease-modifying therapies (DMTs) have redefined AD management strategies and underscore the need to accelerate development of accurate diagnostic procedures to correctly identify patients eligible for these therapies. According to current Appropriate Use Recommendations, confirmation of Aβ pathology is required prior to treatment initiation (Cummings et al., 2023; Rabinovici et al., 2025; Rosen and Jessen, 2025). This can be established through a positive amyloid positron emission tomography (PET) scan based on visual read or via cerebrospinal fluid (CSF) biomarkers consistent with AD pathology. Nevertheless, both amyloid PET imaging and CSF biomarker analysis are costly, not universally accessible, and—in the case of CSF—require an invasive lumbar puncture, limiting their applicability for large-scale screening. As anti-amyloid therapies become more widely implemented in clinical practice, the need to efficiently identify suitable candidates will increase substantially, placing considerable strain on healthcare resources in terms of time, cost and infrastructure. In this scenario, blood-based biomarkers (BBMs) offer a promising alternative (Hampel et al., 2023; Hansson et al., 2023; VandeVrede and Schindler, 2024). Due to their minimally invasive nature, lower cost, and growing evidence supporting their accuracy in detecting cerebral amyloid pathology, BBMs represent a scalable and clinically feasible strategy for early identification of individuals who may benefit from DMTs.

Among the most promising BBMs, Aβ42/Aβ40 has shown highly accurate detection of brain amyloid deposition. In addition, mass-spectrometry (MS) assays have shown superior accuracy compared to most immunoassays (Janelidze et al., 2021).

Here, we present the clinical validation of a predictive model which includes plasma Aβ42/Aβ40 quantified using ABtest-MS, an antibody-free MS-based method, age and apolipoprotein E (APOE) genotype. As previously reported, ABtest-MS has demonstrated strong clinical performance across the AD continuum in controlled research cohorts (Jang et al., 2021; Janelidze et al., 2022; Pascual-Lucas et al., 2023). However, validating biomarkers in real-world cohorts, which encompass greater demographic and clinical diversity, is crucial to establishing their broader applicability in clinical practice for identifying eligible patients for DMTs. This study evaluated the diagnostic accuracy of this predictive model in detecting amyloid pathology in individuals with mild cognitive impairment (MCI) from two real-world cohorts, using approved CSF biomarker measurements as the gold standard for Aβ positivity (Janelidze et al., 2017; Hansson et al., 2018; Keshavan et al., 2021). Furthermore, we aimed to define clinically relevant cutoff values that maximize the probability of accurately detecting brain amyloid deposition to support physicians in their decision-making.

## 2 Methods

### 2.1 Participants in the study

Plasma samples from two different cohorts of participants were used in this validation study. Samples from the Hospital Clínico Universitario San Carlos cohort (HCSC cohort, Madrid, Spain) were used for model training, whereas samples from the Hospital Universitari Santa Maria cohort (HUSM cohort, Lleida, Spain) were used for validation purposes. This clinical validation study was approved by the Ethics Committees of both hospitals. Both were considered ‘real-world’ cohorts as they originated from secondary care specialized memory units.

A total of 450 patients were considered for analysis, with 190 from the HCSC cohort and 260 from the HUSM cohort. In both cases, patients were referred to the memory unit from primary care or other specialized units at HCSC and HUSM, respectively. All patients were diagnosed with MCI according to the recommendations from the National Institute on Aging and Alzheimer’s Association (NIA-AA) workgroups on diagnostic guidelines for AD (Albert et al., 2011).

In this study, the five-year maximum storage time for plasma samples at −80 °C was determined by the longest storage duration within the training cohort (HCSC cohort). To avoid potential bias due to differences in storage time, samples stored for more than 5 years in the validation cohort (HUSM cohort) were excluded from the analysis.

### 2.2 CSF measurements

CSF measurements were performed at the laboratories of the respective hospitals as part of routine clinical practice. In the HCSC cohort, CSF Aβ40, Aβ42, p-tau181 and total tau were analyzed in the Lumipulse® G600 II platform from Fujirebio (Tokyo, Japan). External quality controls (QC) from the University of Gothenburg (The Alzheimer’s Association QC program for CSF and blood biomarkers) were tested quarterly to assure correct assay performance. The cutoff value of Aβ42/Aβ40 that defined CSF-Aβ positivity was ≤ 0.068 (Leitão et al., 2019). In the HUSM cohort, CSF Aβ42, Aβ40 and p-tau181 were also measured with the Lumipulse® G600 II platform. External QC were routinely checked to monitor system performance. In this case, the cutoff value of Aβ42/Aβ40 that defined CSF-Aβ positivity was ≤ 0.069 (Dakterzada et al., 2021). The positivity criteria routinely applied at each center were maintained, given that the differences were marginal (1.4%). For those patients which had no available CSF Aβ42/Aβ40 measures (*n* = 30), a cutoff point of CSF p-tau181/Aβ42 ≥ 0.0815 was used. This value corresponds to the maximum Youden’s Index for CSF p-tau181/Aβ42 after receiver operating characteristic (ROC) analysis in those patients having both measurements (area under the ROC curve, AUC=0.99, data not shown).

### 2.3 Plasma Aβ measurements

Plasma was collected at each center in accordance with its respective standard operating procedures (SOPs). Plasma Aβ40 and Aβ42 were measured using ABtest-MS, an antibody-free high performance liquid chromatography-differential mobility spectrometry-triple quadrupole mass-spectrometry (HPLC-DMS-MS/MS) method developed by Araclon Biotech (Zaragoza, Spain). Briefly, analytes were extracted directly from plasma and no immunoprecipitation procedure was conducted. Intact Aβ40 and Aβ42 species were measured since no enzymatic digestion was performed. Deuterated internal standards (^2^H-Aβ40 and ^2^H-Aβ42, Bachem AG, Bubendorf, Switzerland) were spiked in all samples. Response ratios corresponding to the endogenous species in study samples (^14^N-Aβ40/^2^H-Aβ40 and ^14^N-Aβ42/^2^H-Aβ42) were interpolated into the calibration curves, which were constructed after spiking with the corresponding ^15^N-Aβ analogs. Further details about the analytical procedure and instrumental acquisition parameters are described in the literature (Pannee et al., 2021; Cullen et al., 2021; Janelidze et al., 2022; Allué et al., 2023). Samples from HCSC were analyzed in August 2024, while samples from HUSM were analyzed in October 2024. In accordance with the study design, Araclon Biotech personnel were kept blinded throughout the entire process to sample characteristics and associated information.

### 2.4 APOE genotyping

Genomic DNA was extracted from whole blood using the ReliaPrep Blood gDNA MiniPrep System (Promega, Madison, WI, USA), according to the manufacturer’s instructions. APOE genotype was determined using TaqMan SNP genotyping assays (Thermo, Waltham, MA, USA) targeting rs429358 (position 112) and rs7412 (position 158). Genotyping was performed on a StepOnePlus Real-Time PCR System (Applied Biosystems, Waltham, MA, USA) with TaqMan Genotyping Master Mix, following the manufacturer’s protocol. Positive and negative controls were included in each analytical run for quality control.

### 2.5 Statistical analysis

Statistical analyses and graphical representations of the data were conducted using GraphPad Prism v5.03 (GraphPad Software, San Diego, CA, USA), SPSS v18 (IBM, Armonk, NY, USA) and R statistical software (v 4.4.2, https://www.R-project.org/). To compare different groups, the Chi-square test was used for categorical variables and the Mann–Whitney U test for continuous ones. Bootstrap resampling was performed using the *boot* package in R. Logistic regression and ROC curve analysis was performed with SPSS v18. Linear associations between the biomarker concentrations in plasma and cerebrospinal fluid was estimated using Deming regression in R (*deming* package). Monte Carlo simulations were performed in R to evaluate how the addition of random noise (from 2.5 to 10%) to plasma Aβ42/Aβ40 measurements impacts the classification performance of the predictive model. For each noise level, 1,000 Monte Carlo iterations were generated by perturbing the measurements, refitting the model, and computing the AUC. Model calibration,—specifically, moderate calibration using a flexible calibration curve based on Locally Estimated Scatterplot Smoothing (LOESS)— was also carried out in R with the *val.prob.ci.2* function from the *CalibrationCurves* package, in order to assess concordance between predictions and observed proportions. A two-tailed *p* value of <0.05 was considered statistically significant.

## 3 Results

### 3.1 Demographic characteristics

The baseline characteristics of HUSM and HCSC cohorts divided according to CSF-amyloid status are summarized in Table 1. In the HUSM cohort, individuals with CSF-Aβ(+) status were older than their CSF-Aβ(−) counterparts (median [interquartile range, IQR]: 74.0 [71.0–77.0] vs. 70.0 [65.0–74.3]; *p* < 0.0001) and showed lower performance on the Mini-Mental State Examination (MMSE: 26.0 [24.0–27.8] vs. 27.0 [24.3–28.0]; *p* = 0.020). Gender distribution did not differ significantly between groups (*p* = 0.213), while APOE ε4 allele distribution was significantly different (*p* < 0.0001).

**Table 1:** Baseline characteristics of the study participants.

Characteristic	HUSM			HCSC		
	CSF-Aβ(+) (*n* = 182)	CSF-Aβ(−) (*n* = 78)	*p*-value	CSF-Aβ(+) (*n* = 96)	CSF-Aβ(−) (*n* = 94)	*P*-value
Age, years	74.0 [71.0–77.0]	70.0 [65.0–74.3]	**<0.0001**	72.5 [68.3–76.0]	66.5 [61.0–72.0]	**<0.0001**
Female, No. (%)	116 (63.7)	43 (55.1)	0.213	57 (59.4)	49 (52.1)	0.381
MMSE, score	26.0 [24.0–27.8]	27.0 [24.3–28.0]	**0.020**	27.0 [25.0–29.0]	28.0 [26.0–30.0]	**0.001**
APOE ε4 carriers, No. (%)	104 (57.1)	11 (14.1)	**<0.0001**	53 (55.2)	18 (19.1)	**<0.0001**
APOE ε4, No. (%)			**<0.0001**			**<0.0001**
0 allele ε4	78 (42.9)	67 (85.9)		43 (44.8)	76 (80.9)	
1 allele ε4	91 (50.0)	9 (11.5)		42 (43.8)	18 (19.1)	
2 alleles ε4	13 (7.1)	2 (2.6)		11 (11.5)	0 (0)	
Sample storage time, years	1.5 [0.9–2.2]	1.5 [0.8–2.3]	0.910	0.8 [0.33–1.3]	0.6 [0.4–1.3]	0.878
Plasma Aβ40, pg/mL	226.3 [199.9–255.9]	226.0 [206.1–265.1]	0.230	233.9 [206.4–264.2]	216.7 [201.2–252.2]	0.144
Plasma Aβ42, pg/mL	44.0 [38.1–50.4]	50.9 [45.3–58.6]	**<0.0001**	43.0 [37.6–48.2]	47.6 [42.3–53.4]	**<0.0001**
Plasma Aβ42/Aβ40	0.195 [0.180–0.212]	0.221 [0.209–0.240]	**<0.0001**	0.185 [0.174–0.204]	0.216 [0.198–0.227]	**<0.0001**
CSF p-tau181, pg/mL	94.0 [69.1–127.5]	39.5 [29.7–48.3]	**<0.0001**	96.2 [73.4–143.2]	38.8 [29.5–50.3]	**<0.0001**
CSF t-tau, pg/mL	581.0 [444.0–769.5]	249.0 [192.0–332.5]	**<0.0001**	635.5 [473.8–863.3]	281.0 [215.0–367.3]	**<0.0001**
CSF Aβ40, pg/mL	10726.0 [8147.0–13469.0]	10799.0 [8047.3–12982.0]	0.996	13384.0 [10552.3–16648.8]	12405.0 [9258.5–15813.3]	0.131
CSF Aβ42, pg/mL	438.5 [327.8–581.8]	917.5 [725.3–1233.3]	**<0.0001**	641.0 [510.0–779.5]	1280.0 [937.8–1686.8]	**<0.0001**
CSF Aβ42/Aβ40	0.043 [0.035–0.049]	0.089 [0.081–0.102]	**<0.0001**	0.049 [0.042–0.058]	0.109 [0.099–0.114]	**<0.0001**
CSF p-tau181/Aβ42	0.216 [0.149–0.321]	0.041 [0.032–0.051]	**<0.0001**	0.150 [0.108–0.246]	0.029 [0.025–0.035]	**<0.0001**

HUSM, Hospital Universitari Santa Maria; HCSC, Hospital Clínico Universitario San Carlos; APOE, apolipoprotein E; MMSE, Mini-Mental State Examination; CSF, cerebrospinal fluid; Aβ, Amyloid-beta; p-tau181, phosphorilated protein Tau at threonine 181. Data are median and interquartile range [IQR] values, except for the variables female and APOE (number of alleles or dichotomized) which are the number of cases (%). Differences between groups were tested using Mann–Whitney and Chi-square tests, as appropriate. *p*-values in bold correspond to statistically significant results.

Similarly, in the HCSC cohort, CSF-Aβ(+) individuals were older than their CSF-Aβ(−) counterparts (72.5 [68.3–76.0] vs. 66.5 [61.0–72.0]; *p* < 0.0001) and performed worse on the MMSE (27.0 [25.0–29.0] vs. 28.0 [26.0–30.0]; *p* = 0.001). Gender distribution remained comparable (*p* = 0.381), whereas the distribution of APOE ε4 alleles differed significantly between groups (*p* < 0.0001). The prevalence of CSF-Aβ positivity differed between cohorts: 70% in the HUSM cohort compared to 51% in the HCSC cohort. In both cohorts, significant differences were observed in plasma and CSF biomarker values between CSF-Aβ(+) and CSF-Aβ(−) individuals, except for Aβ40. Supplementary Table 1 shows baseline characteristics by cohort.

### 3.2 Clinical performance in the identification of CSF-Aβ(+) individuals

HCSC cohort: a moderate positive association was observed between plasma and CSF Aβ42/Aβ40 levels (Spearman’s rho = 0.524, *p* < 0.0001). Deming regression yielded a slope of 0.74, suggesting that increases in plasma levels were accompanied by proportional increases in CSF levels (Supplementary Figure 1a). Plasma Aβ42/Aβ40 was 12.6% lower in CSF-Aβ(+) individuals (*p* < 0.0001) and yielded an AUC after ROC curve analysis of 0.81 (95% confidence interval [CI], 0.75–0.87) for the identification of CSF-Aβ status (Supplementary Table 2). The inclusion of the rest of the covariates, age and presence of APOE ε4 copies (dichotomized), produced a marked decrease in Akaike Information Criterion (AIC) value yielding a final AUC of 0.89 (95% CI, 0.84–0.93) and an accuracy of 82.3%.

In HUSM cohort, plasma and CSF levels were also positively associated (Spearman’s rho 0.470, *p* < 0.0001). Deming regression slope was 1.24 (Supplementary Figure 1b). Plasma Aβ42/Aβ40 was 12.9% lower in CSF-Aβ(+) individuals (*p* < 0.0001) and yielded an AUC value of 0.80 (95% CI, 0.75–0.86) after ROC analysis. Again, introduction of age and APOE genotype, produced a significant decrease of AIC value (Supplementary Table 2) yielding a final AUC of 0.88 (95% CI, 0.84–0.93) and an accuracy of 81.6%. Table 2 shows the relevant metrics obtained from the analysis of both cohorts separately. All the metrics are calculated at the maximum Youden’s Index.

**Table 2:** Model performance metrics.

Cohort	Sensitivity	Specificity	PPV	NPV	AUC	Accuracy (%)	Prevalence (%)	*N*
HCSC (T)	0.92	0.71	0.77	0.89	0.89	82.3	51	190
HUSM (V)	0.81	0.85	0.93	0.66	0.88	81.6	70	260
Ext-Val	0.83	0.81	0.91	0.67	0.88	82.3	70	260
Combined	0.82	0.80	0.87	0.73	0.88	81.1	62	450

HUSM, Hospital Universitari Santa Maria; HCSC, Hospital Clínico Universitario San Carlos; (T), Training cohort; (V), Validation cohort; Ext-Val, External validation; Combined, HUSM+HCSC; PPV, Positive Predictive Value; NPV, Negative Predictive Value; AUC, Area Under the ROC Curve; *N*, number of participants. All the metrics are calculated at the maximum Youden index.

Next, we performed an external validation analysis. Regression coefficients (β_1_ to β_4_) corresponding to model covariates plus the intercept, obtained after the analysis of HCSC data (training cohort) were applied to HUSM participant data (validation cohort). Table 2 shows the metrics obtained in this external validation exercise. Some differences are observed in certain performance metrics between cohorts, mainly due to the large difference in CSF-Aβ positivity (51 vs. 70%). An AUC value of 0.88 (95% CI, 0.84–0.91) and an accuracy of 82.3% were achieved in the validation cohort.

**Figure 1:**
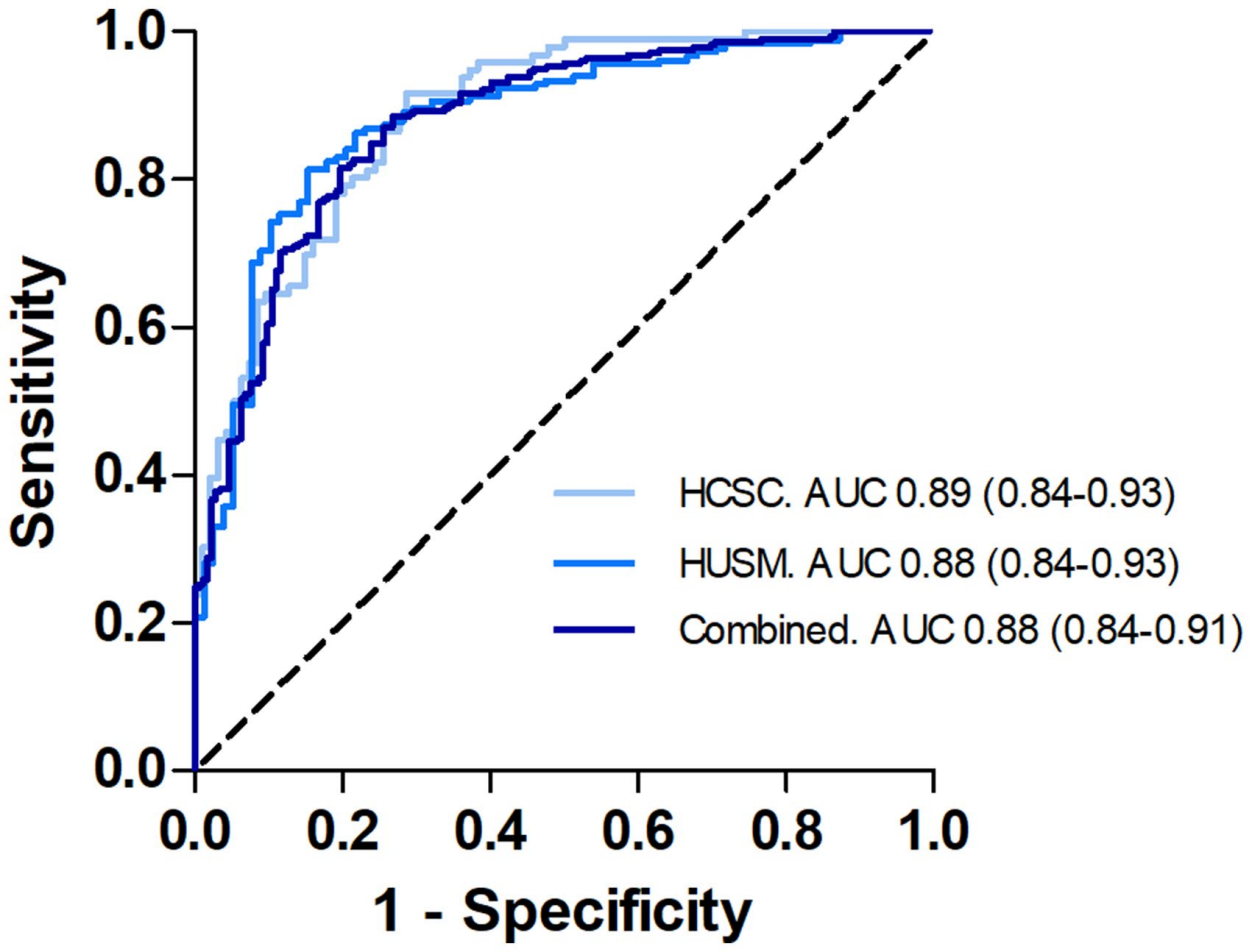
ROC curves obtained after the analysis of each cohort separately and after combination.

**Figure 2:**
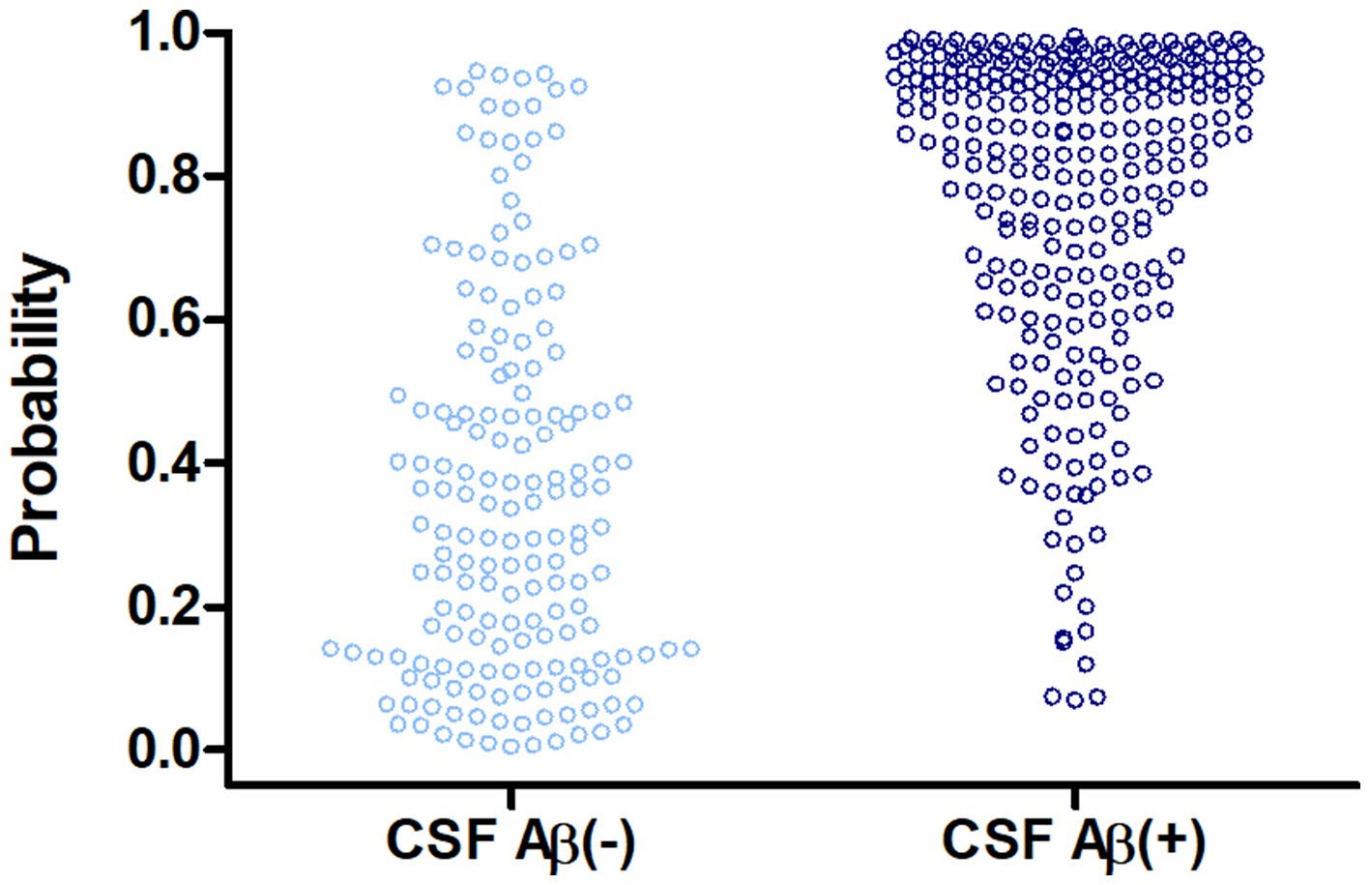
Distribution of model predicted probabilities after combined analysis (450 participants).

Figure 1 shows the ROC curves obtained after separate and combined analysis of both cohorts. The analysis of the combined cohorts yielded an AUC value of 0.88 (95% CI, 0.83–0.92) and an overall accuracy of 81.1% for the predictive model (Table 2). The regression model did not account for information regarding the cohort of origin of the samples. CSF-Aβ positivity prevalence in this combined analysis was 62%. The positive and negative predictive values (PPV and NPV, respectively) varied in the expected direction according to prevalence, as anticipated given the known dependence of these metrics on disease prevalence (pre-test probability). Figure 2 shows the distribution of predicted probabilities after combined analysis, by CSF-Aβ status.

**Figure 3:**
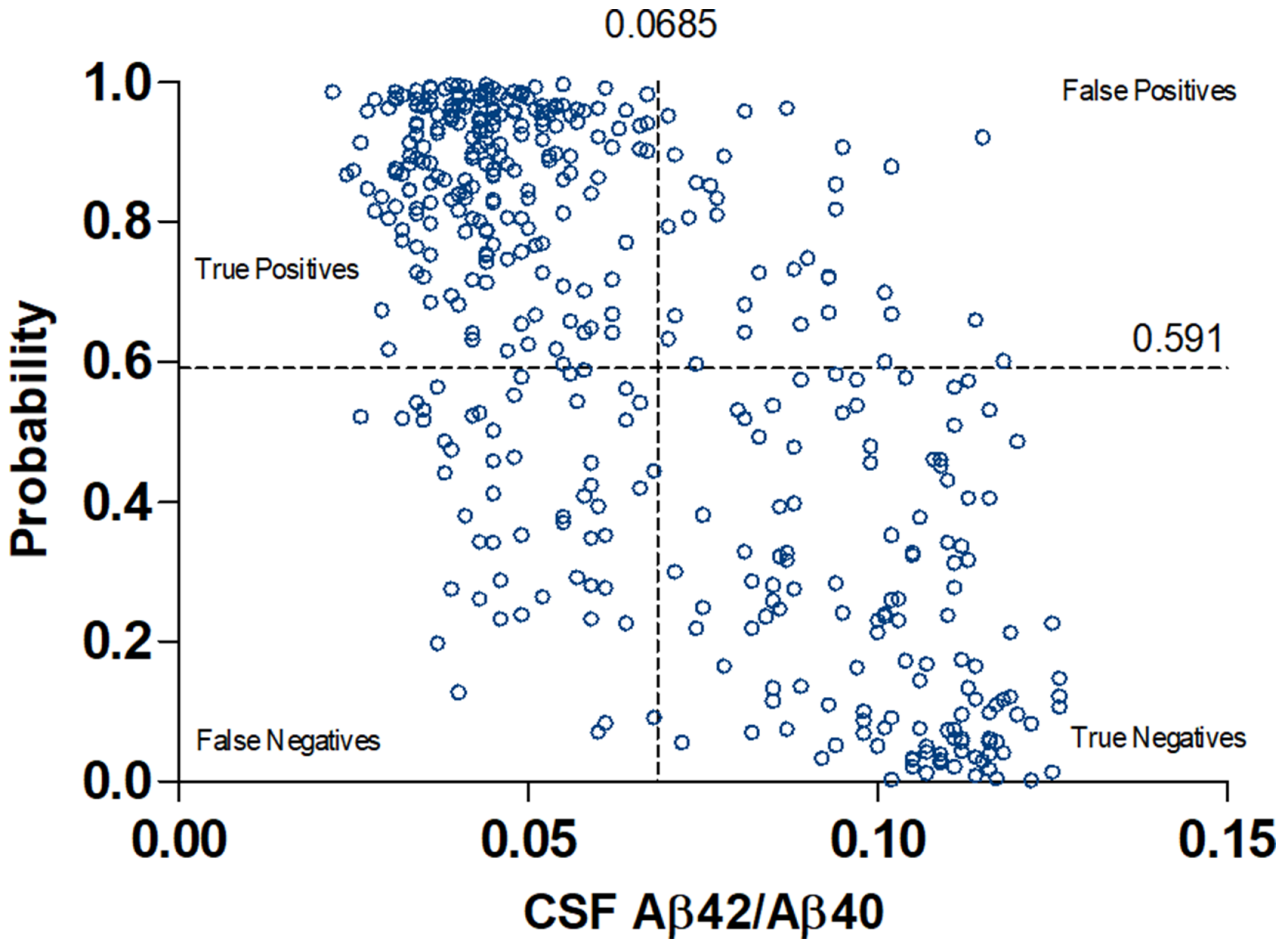
Concordance plot between predicted probabilities and CSF Aβ42/Aβ40 values. A single probability cutoff (calculated at the maximum Youden Index) of 0.591 was used. A mean value of 0.0685 was used to define CSF positivity (average of cutoff values used in each cohort separately).

The concordance plot between predicted probabilities and CSF Aβ42/Aβ40 values for those individuals with available Aβ42/Aβ40 measurements (420 out of 450, since CSF-Aβ positivity was based on p-tau181/Aβ42 in 30 individuals) is shown in Figure 3. The number of false negatives (51, 11.3%) is slightly higher than false positives (34, 7.5%), using a single cutoff value (Youden’s maximum).

**Figure 4:**
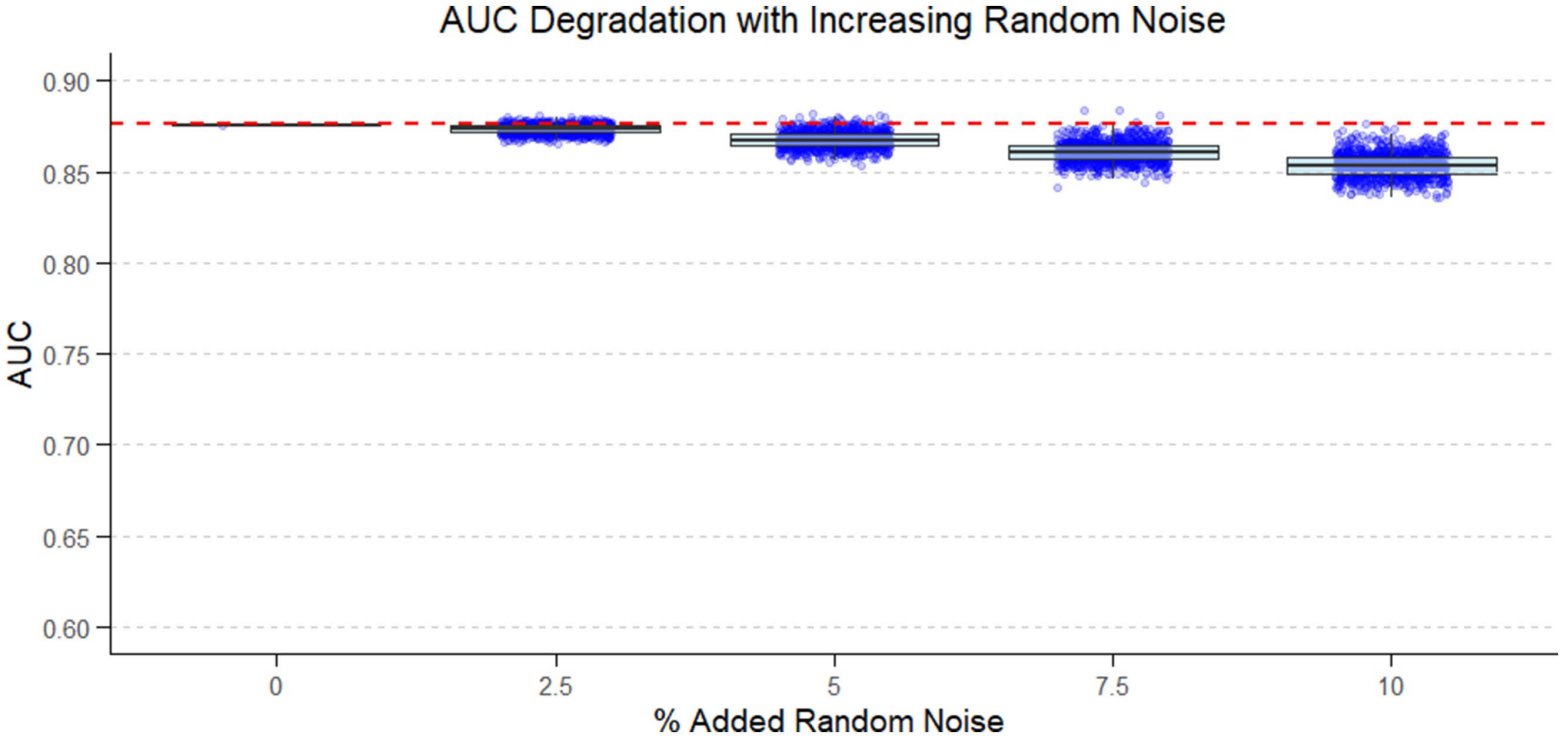
Boxplots with individual data points generated to visualize the distribution of AUC values across noise levels. Red dashed line indicates the original analysis without adding extra noise. Boxes represent the interquartile range (IQR), the line indicates the median, and whiskers extend to 1.5 × IQR.

To evaluate the robustness of our predictive model against additional and unknown sources of variability, we conducted Monte Carlo simulations introducing random measurement noise into the plasma Aβ42/Aβ40 ratio in the combined cohort. The original AUC of 0.88 corresponding to the predictive model, already reflects the intrinsic variability of the plasma Aβ42/Aβ40 measurements, including random and systematic errors introduced from sample extraction to LC–MS analysis. In our simulation, the additional noise levels of 2.5, 5, 7.5, and 10% were superimposed on top of this inherent variability, effectively modeling a scenario in which the analytical assay performs with progressively lower precision. As shown in Table 3 and Figure 4, the introduction of additional noise had no meaningful effect on the AUC of the predictive model.

**Table 3:** Model performance (AUC) after random noise addition to Aβ42/Aβ40 measurements.

Added noise (%)	AUC (Mean)	SD	ΔAUC
Original	0.88	-	-
2.5	0.87	0.002414	−0.0022
5.0	0.87	0.004489	−0.0080
7.5	0.86	0.005656	−0.0148
10.0	0.85	0.006611	−0.0220

SD, standard deviation. Symbols; Δ: increment. AUC values rounded to two decimal figures.

**Figure 5:**
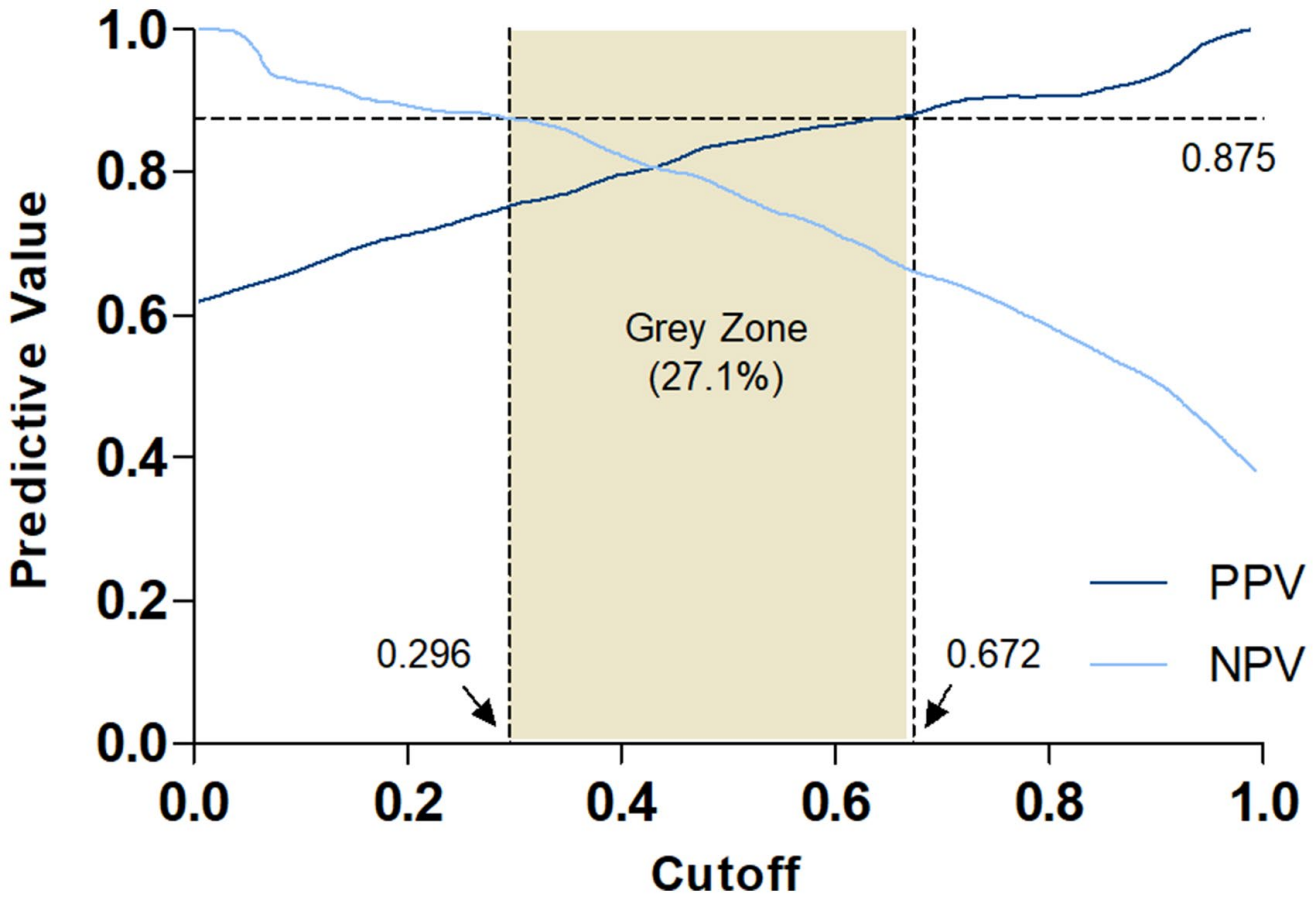
Evolution of PPV and NPV along the probability range. Both predictive values were fixed at 87.5% (black horizontal dashed line). According to this graph, 27.1% of the patients would fall in the grey zone (0.30 ≤ *p* ≤ 0.67) and would need additional testing.

Once combined analysis was performed, PPV and NPV of 87.5% were chosen in order to stablish two cutoff points. This value of 87.5% is a good trade-off between high accuracy and a minimized number of individuals remaining in the uncertainty or grey zone. For these individuals, additional testing, such as lumbar puncture for CSF biomarker analysis or Aβ-PET scans, should be necessary in order to confirm or discard Aβ pathology. The evolution of PPV and NPV along the whole range of probability values (0 to 1) is shown in Figure 5. Setting both predictive values at 87.5% generates two cutoffs, a negativity cutoff at *p* = 0.296 and a positivity cutoff at *p* = 0.672 (rounded at 0.30 and 0.67, respectively) for a final prevalence of 62%. According to this, individuals with probability values ≤ 0.30 have a low likelihood of amyloid plaques and individuals with probability values ≥ 0.67 have a high likelihood of amyloid plaques. Those individuals with intermediate probability (0.30 < *p* < 0.67) fall into the uncertainty or grey zone.

After exclusion of subjects in the grey zone (27%) a sensitivity of 94.4% and specificity of 74.3% were obtained. In order to study model performance in different prevalence scenarios and starting from the combined sample (450 individuals and 62% of prevalence), 1,000 random populations were generated with bootstrap analysis at fixed prevalence values of 40, 50 and 60% (3.000 populations in total). The results of these analyses are shown in Table 4. Again, predictive values of 87.5% were fixed and metrics calculated after excluding subjects falling in the grey zone. For a prevalence value of 60%, selected for practical purposes according to published references for the MCI population (Janssen et al., 2021; Hu et al., 2022), results must be interpreted as follows: *p* ≤ 0.30 (30%) indicates low probability of brain amyloid plaques, *p* ≥ 0.69 (69%) indicates high probability of amyloid plaques and intermediate probability (0.30 < *p* < 0.69, between 30 and 69%) indicates an indeterminate result. These results show that more than 70% of invasive and expensive tests could be avoided while maintaining a high classification accuracy. Alternative results after setting predictive values at 85% are shown in Supplementary Table 3 and Supplementary Figure 2. For a prevalence of 60%, the proportion of individuals falling within the grey zone decreases to 15.3%, with sensitivity and specificity remaining comparable to the previous scenario (predictive values of 87.5%), albeit with a slightly reduced overall accuracy (85%).

**Table 4:** Model performance metrics in the simulated populations.

Prevalence (%)	Original	Bootstrap [Mean (SD)]		
	62	40	50	60
Lower cutoff	0.296	0.545 (0.08)	0.413 (0.09)	0.299 (0.19)
Upper cutoff	0.672	0.922 (0.03)	0.858 (0.12)	0.686 (0.08)
Subjects in intermediate zone (%)	27.1	35.3 (9.1)	35.3 (16.3)	28.3 (15.6)
Sensitivity (%)	94.4	70.3 (6.4)	85.3 (4.6)	93.9 (2.6)
Specificity (%)	74.3	95.3 (1.4)	89.4 (3.4)	76.2 (9.6)

All the metrics are calculated after exclusion of individuals falling in the grey zone. SD, standard deviation.

**Figure 6:**
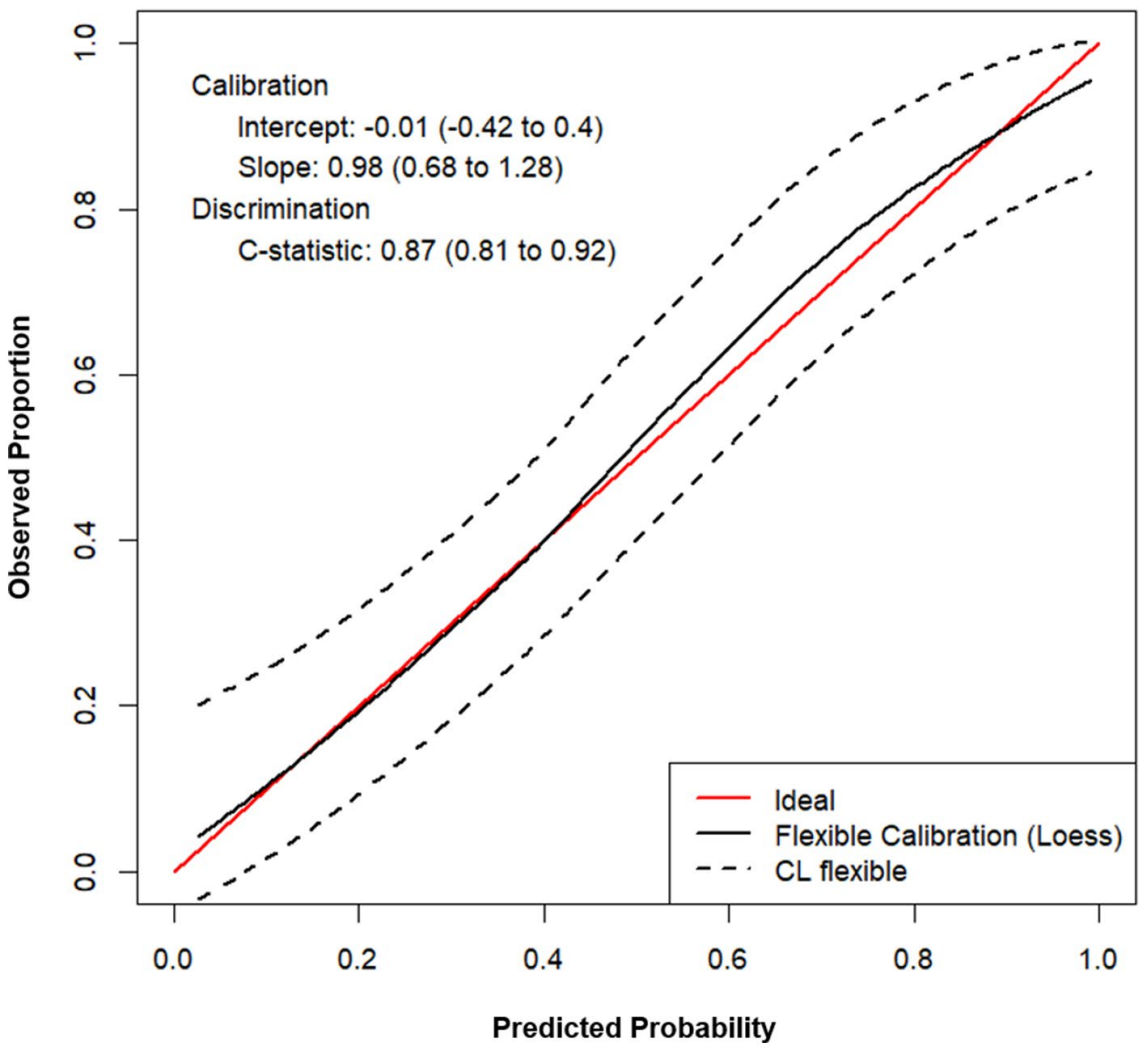
Average calibration plot after 1.000 bootstrap iterations. Flexible calibration line approaches ideal calibration line (Slope = 0.98 and Intercept = −0.01) indicating good calibration.

Finally, model calibration (Van Calster et al., 2016; Riley et al., 2024) was performed to assess the agreement between model predictions and observed proportions. Due to sample size limitations, we conducted 1.000 bootstrap cycles, performing calibration at each iteration. A population with a prevalence of 60% was considered in the calculations. The average calibration curve is shown in Figure 6. The slope and intercept values (0.98 and −0.01, respectively) are very close to their target values (1 and 0, respectively), and the flexible calibration curve closely approximates the ideal calibration line indicating that the model is, on average, properly calibrated.

## 4 Discussion

In this work, we have clinically validated a predictive model composed of plasma Aβ42/Aβ40, age and APOE genotype (presence or absence of APOE ε4 alleles) to infer the brain amyloid status of individuals with MCI. A distinctive feature of this study was that Aβ40 and Aβ42 were quantitated using ABtest-MS. This analytical procedure is based on antibody-free direct extraction from plasma, followed by detection and quantitation of both intact peptides by micro-HPLC coupled to DMS, a variant of ion mobility spectrometry, and triple quadrupole tandem mass spectrometry (MS/MS). In previous studies, ABtest-MS has demonstrated good clinical performance across the AD continuum (Jang et al., 2021). As an example, we identified brain amyloid status (as determined by ^18^F-Florbetaben PET) with high accuracy in a well-characterized cohort of individuals with subjective cognitive decline (SCD), and externally validated these results in a completely independent cohort (Pascual-Lucas et al., 2023). In this work, we have extended these findings to the MCI population, specifically, to two real-world clinical cohorts from two specialized memory units.

In this study, ABtest-MS has demonstrated a high predictive ability to identify CSF-Aβ(+) individuals from two real-world cohorts. Validating diagnostic methods in real-world cohorts provides a significant advantage by evaluating their performance in diverse, clinically representative populations, thus improving the generalizability and translational applicability of the results (Schöll et al., 2024). Unlike well-characterized convenience cohorts, which typically consist of participants with more homogeneous and clear-cut diagnostic characteristics, real-world cohorts better capture the complexity and variability of typical clinical practice. These cohorts include individuals with a broader spectrum of comorbidities and atypical clinical presentations. Such diversity allows for a more accurate reflection of how diagnostic tools will perform in routine healthcare settings. However, this comes with notable challenges, that are reflected in our study cohorts. Both cohorts were drawn from real-world secondary-care MCI populations assessed with comparable diagnostic procedures, following the recommendations from the NIA-AA workgroups on diagnostic guidelines for AD (Albert et al., 2011), which supports the use of one for model training and the other for independent validation. At the same time, relevant differences were observed between cohorts: the HUSM cohort was older, had lower MMSE scores, and—most importantly—showed a higher prevalence of CSF-amyloid positivity. These contrasts indicate that the two cohorts capture somewhat different stages or referral patterns within secondary care, which strengthens the robustness of the validation design by testing the model in real routine clinical practice settings.

Previous studies have highlighted the vulnerability of the Aβ42/Aβ40 ratio in plasma to minor measurement or pre-analytical deviations, which can easily lead to misclassifications due to the modest fold-change between amyloid-positive and negative individuals (Rabe et al., 2023). In contrast, our Monte Carlo simulations showed that the multivariable predictive model—combining the Aβ42/Aβ40 ratio with age and APOE genotype—not only achieves higher classification accuracy but is also remarkably resilient to random, largely unknown, and inherently uncontrollable sources of variability. Even after artificially introducing up to 10% additional random noise into the ratio, the model’s AUC decreased only marginally, reflecting its intrinsic capacity to buffer these unpredictable fluctuations. This robustness provides a strong rationale for using the multivariable model instead of the crude biomarker, as it both improves diagnostic performance and mitigates the impact of unavoidable variability in real-world conditions.

A target prevalence of 60% was selected, based on prior work (Janssen et al., 2021; Hu et al., 2022). This value is almost identical to the one obtained after combined analysis. Additionally, cutoff values at different prevalence values are explored, so they can be applied to different clinical settings (DeMarco et al., 2024). Using two cutoff points enhances diagnostic precision by reducing uncertainty in borderline cases (Schindler et al., 2024), therefore we adopted this approach. These findings suggest that implementing this diagnostic approach could significantly reduce the reliance on invasive and costly procedures, with over 70% potentially being avoided. Importantly, this reduction does not come at the expense of diagnostic performance, as high classification accuracy is maintained. Finally, we checked model calibration. Well-calibrated models, as in this case, are far more valuable in clinical practice as they provide realistic risk estimations even if the AUC of the model is lower than a non-properly calibrated model with higher AUC value (Van Calster et al., 2019). Importantly, a well-calibrated predictive model provides probability estimates that reflect the actual risk of amyloid positivity, allowing neurologists and other dementia specialists to make more informed decisions with greater confidence. In this work, we have extensively evaluated the clinical performance of the method, ensuring a comprehensive analysis by considering different prevalence values and establishing various predictive power thresholds. This approach has allowed us to explore how changes in prevalence and predictive power can affect the clinical utility of the test, providing insights into its effectiveness and potential limitations under a range of real-world conditions.

Our logistic model based on plasma Aβ42/Aβ40 demonstrates strong diagnostic performance under clinically realistic conditions. By fixing both positive and negative predictive values at 87.5%, every classified individual receives a test result with high confidence (87.5% accuracy), while indeterminate cases are excluded from accuracy calculations, as is standard in a dual cutoff strategy. The predictive value of biomarker tests varies according to the prevalence of amyloid positivity and must always be interpreted within the full clinical context (Schindler et al., 2024), as predictive values directly reflect the post-test probability of disease for an individual patient, whereas sensitivity and specificity are intrinsic test characteristics that provide no direct estimate of a patient’s likelihood of disease. When evaluated at a standard prevalence of 60%, commonly found in MCI populations, the model’s performance aligns with the recent Global CEO Initiative on Alzheimer’s Disease recommendations for triaging tests and approaches the 90% PPV and NPV for confirmatory testing (Schindler et al., 2024). Nevertheless, these guidelines also recommend a grey zone <20%. While our model exceeds this threshold, in clinical practice, physicians accept a grey zone larger than 20% if it translates into higher accuracy and confidence for the individuals who do receive a test result, particularly in heterogeneous, real-world cohorts where indeterminate outcomes are naturally more frequent than in well-controlled populations (Sarto et al., 2025). Consistent with this, similar or higher grey-zone rates are frequently reported in other studies with biomarkers such as p-tau217 that still achieve the 90% sensitivity and specificity criteria (Arranz et al., 2024; Álvarez-Sánchez et al., 2025; Wilson et al., 2025; Rudolph et al., 2025). Overall, these findings reinforce the robustness of the model and its ability to deliver highly reliable classifications in clinically relevant scenarios.

For a biomarker to be translated into clinical practice, it must be demonstrated that performance observed in well-characterized discovery cohorts also holds across heterogeneous real-world populations. The results presented here are in good agreement, in terms of classification accuracy, with previous studies using our model in discovery cohorts such as DPUK-Korea and FACEHBI (Jang et al., 2021; Pascual-Lucas et al., 2023), despite differences in cohort composition, including diagnostic groups and prevalence rates. The consistent predictive performance observed in these real-world settings further supports the clinical utility of the model. Importantly, our approach offers several practical advantages: according to several recent publications, the plasma Aβ42/Aβ40 ratio is not significantly affected by renal function or BMI, factors known to influence other biomarkers such us p-tau217 (Syrjanen et al., 2022; Pichet Binette et al., 2023; Lehmann et al., 2023; Lehmann et al., 2024; Piura et al., 2025). In addition, the logistic regression model inherently adjusts for age and APOE genotype, thus eliminating the need for age-stratified interpretation. Altogether, these findings support the use of our model as a reliable and scalable strategy for estimating brain amyloid status, while deferring ambiguous cases to additional confirmatory evaluation (e.g., PET or CSF analysis).

This study has several limitations. While the number of participants cannot be considered small (*n* = 450), a larger sample size could further enhance the generalizability of the findings. A larger and more diverse cohort would allow for a broader representation of different populations and potential subgroups, helping to confirm whether the results could be consistently applied across diverse settings. Although the current sample provides valuable insights, future studies with larger, more ethnically diverse cohorts would further strengthen the external validity of the conclusions. Additionally, prospective validation of any predictive model is essential, and further studies are planned for this purpose.

A further limitation is the use of a single BBM, which reinforces the rationale for integrating additional plasma biomarkers into the model to potentially increase its predictive accuracy. Among the different available BBMs, p-tau217 has shown high performance and its combination with Aβ42/Aβ40 (Meyer et al., 2024) may further enhance accuracy in future applications, as it often yields the numerically strongest models and improves overall model fit (e.g., AIC, BIC), reflecting the biological complementarity of these markers beyond what is captured by AUC values alone.

Exploring the contribution of other markers will be addressed in future studies.

## 5 Conclusion

We have demonstrated strong clinical performance of a predictive model based on Aβ42/Aβ40 ratio measurements obtained through ABtest-MS, for identifying brain amyloid deposition in individuals with MCI. Cutoff values for positive and negative results were determined across different prevalence scenarios, while ensuring high predictive accuracy. In a real-world population with a 60% CSF-Aβ(+) prevalence, more than 70% of invasive and costly tests could be avoided, facilitating rapid and cost-effective identification of candidates for DMTs.

## Data Availability

The raw data supporting the conclusions of this article will be made available by the authors upon reasonable request, in accordance with institutional and ethical requirements.
