# Supplementary material for: Clinical validation of a plasma-based antibody-free LC–MS method for identifying CSF amyloid positivity in mild cognitive impairment
Source: Front Aging Neurosci. 2025 Oct 10;17:1681516. doi: 10.3389/fnagi.2025.1681516 (PMC12549660; doi:10.3389/fnagi.2025.1681516)
Supplement: Supplementary file 1 [file Data_Sheet_1.DOCX]

**Supplementary Figure 1.-** Associations between Aβ42/Aβ40 in plasma and CSF.

**A.- HCSC Cohort.** Deming regression. Slope = 0.739 (95% CI, 0.594-0.883), Intercept = 0.144 (95% CI, 0.132-0.155).


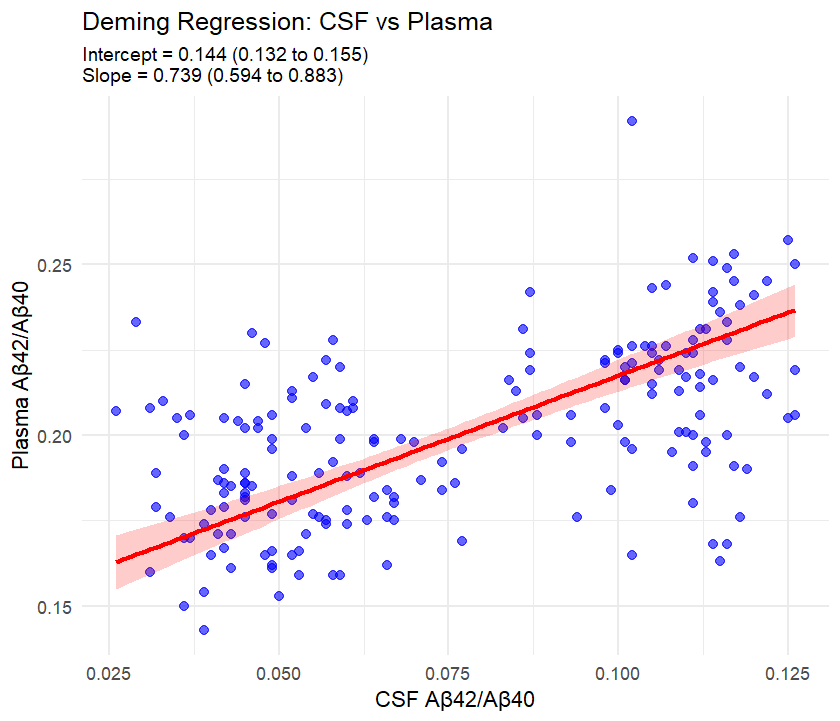


**B.- HUSM Cohort.** Deming regression. Slope = 1.239 (95% CI: 0.984-1.494), Intercept = 0.136 (95% CI, 0.122-0.149).


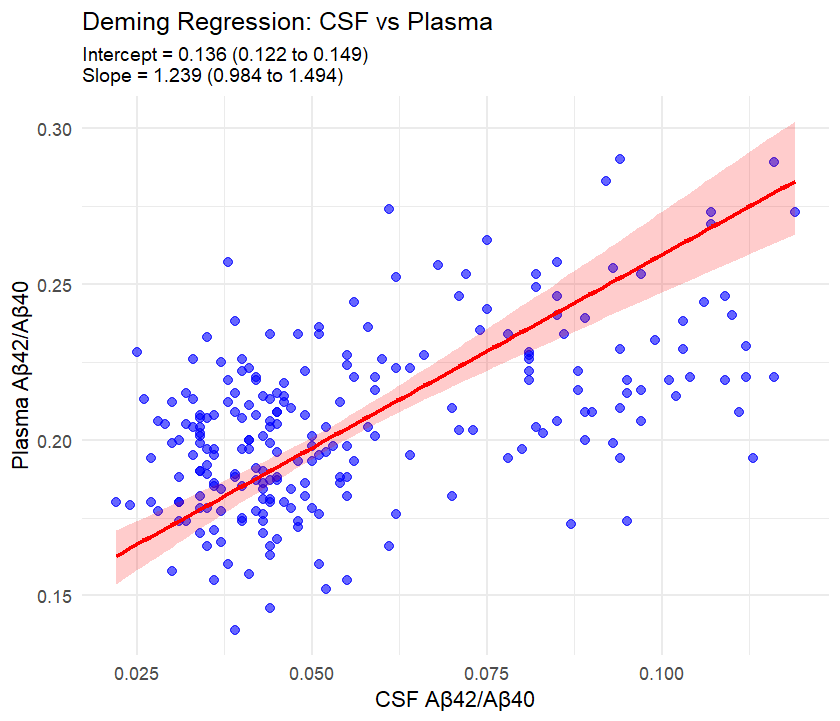


**Supplementary Figure 2.-** Estimated cutoffs for a population with a CSF-Aβ positivity prevalence of 60% after setting both negative and positive predictive values (NPV and PPV) at 85%.


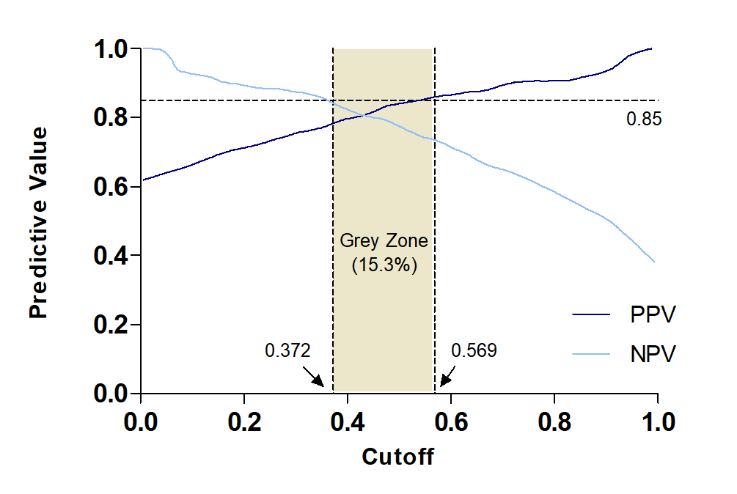


**Supplementary Table 1.** Baseline characteristics of the study participants by cohort.

Abbreviations; HUSM: Hospital Universitari Santa Maria; HCSC: Hospital Clínico Universitario San Carlos; APOE: apolipoprotein E; MMSE: Mini-Mental State Examination; CSF: cerebrospinal fluid; Aβ: Amyloid-beta; p-tau181: phosphorilated protein Tau at threonine 181. Data are median and interquartile range [IQR] values, except for the variables female, APOE (number of alleles or dichotomized) and CSF status, which are the number of cases (%). Differences between groups were tested using Mann-Whitney and Chi-square tests, as appropriate.

**Supplementary Table 2. –** Predictive model diagnostics.

**HCSC cohort**

| **Model** | **R^2^ (Nagelkerke)** | **AIC** | **AUC (95% CI)** |
| --- | --- | --- | --- |
| Aβ42/Aβ40 | 0.383 | 193.97 | 0.81 (0.75-0.87) |
| Age + APOE | 0.392 | 203.19 | 0.82 (0.76-0.88) |
| Aβ42/Aβ40 + Age + APOE | 0.553 | 169.52 | 0.89 (0.84-0.93)* |

**HUSM cohort**

| **Model** | **R^2^ (Nagelkerke)** | **AIC** | **AUC (95% CI)** |
| --- | --- | --- | --- |
| Aβ42/Aβ40 | 0.326 | 253.76 | 0.80 (0.75-0.86) |
| Age + APOE | 0.316 | 258.14 | 0.80 (0.74-0.85) |
| Aβ42/Aβ40 + Age + APOE | 0.500 | 212.51 | 0.88 (0.84-0.93)* |

Abbreviations: HCSC, Hospital Clínico Universitario San Carlos; HUSM, Hospital Universitari Santa Maria; AIC, Akaike´s information criterion; APOE, apolipoprotein E; AUC, area under the ROC curve; CI, confidence interval.

* AUC significantly higher than that of the demographic model (APOE and age), as determined by DeLong’s test (P = 0.0015 and 0.0001 for HCSC and HUSM cohorts, respectively).

**Supplementary Table 3.** Model performance metrics in the simulated populations after setting positive and negative predictive values (PPV and NPV) at 85%.

|  | **Original** | **Bootstrap [Mean (SD)]** | | |
| --- | --- | --- | --- | --- |
| **Prevalence (%)** | 62 | 40 | 50 | 60 |
| **Lower cutoff** | 0.363 | 0.623 (0.05) | 0.493 (0.07) | 0.372 (0.08) |
| **Upper cutoff** | 0.545 | 0.898 (0.06) | 0.750 (0.15) | 0.569 (0.11) |
| **Indetermediate (%)** | 14.0 | 26.7 (10.9) | 21.1 (15.1) | 15.3 (12.9) |
| **Sensitivity (%)** | 93.2 | 67.5 (6.6) | 83.8 (3.6) | 92.3 (1.6) |
| **Specificity (%)** | 70.3 | 93.9 (1.8) | 86.1 (3.0) | 73.0 (4.3) |

Abbreviations; SD: standard deviation.
